# The Care of Adults with Intellectual Disabilities: Informal (Family) Caregivers’ Perspectives

**DOI:** 10.3390/ijerph192315622

**Published:** 2022-11-24

**Authors:** Anna Gutowska

**Affiliations:** Department of Andragogy and Social Gerontology, University of Lodz, 90-136 Łodź, Poland; anna.gutowska@now.uni.lodz.pl

**Keywords:** care, caregiver, intellectual disability, adult with an intellectual disability

## Abstract

Context: The care of adults with intellectual disabilities is marginalized and rarely studied in Poland. In recent years, this issue has gained particular importance, partly due to the increasing life expectancy of people with ID. This paper presents a study of the function of informal caregivers for adults with ID, comprising parents who provide regular, constant, physical and emotional support and assistance with everyday activities to their adult children. Due to cultural and institutional conditions, Polish society sets high expectations for families regarding the care of their dependent members. Social policy also mainly promotes informal care, with formal care only being supported to a very limited extent. The state delegates responsibility, including financial responsibility, to families. With the rapid aging of society, this situation poses great challenges. Methods: This study was conducted in the Łodź region of Poland; it used a qualitative approach, and a semi-structured interview was performed using the narrative elements technique. The main goal of the research was to understand the situation of caregivers to adults with an intellectual disability by identifying thematic categories in the respondents’ statements. The analysis of the qualitative data content made it possible to capture and present the participants’ personal perspectives on significant issues connected with their function in the context of providing care to an adult with an intellectual disability. A total of 12 interviews were conducted. The age of the respondents (caregivers) was 51–82 years old, and the individuals they were caring for were between 20 and 49 years old. Results: Based on the materials collected, 13 thematic categories and subcategories were identified, along with illustrative examples. The main categories concerned everyday functioning, health, uncertainty, relationships with others, feelings, time, and the macro level. For each category, subcategories were distinguished and illustrated by the respondents’ statements. The categories and subcategories were not completely distinct; sometimes they overlapped or complemented one another. Conclusions: For the majority of the respondents, the care of an adult with an intellectual disability had a negative effect on their well-being. As a consequence, they performed their caregiver’s role at the expense of their own lifestyle. Noticeable themes included “addiction” to caregiving, psychophysical fatigue, and the needs and difficulties resulting from this being “ignored” by the commonly understood social environment (including state institutions). Thus, the care of dependent adults with ID should be viewed on a broad human spectrum, that is, in consideration of the unique situation of those who remain under permanent care provided by family members, those who live alone, and those whose loved ones try to combine caregiving with their own private lives. This is becoming all the more important, as the number of seniors with intellectual disabilities will continue to grow in the coming decades.

## 1. Introduction

Some adults with intellectual disabilities possess limited independence. (We could not obtain data about the number of people with disabilities in Poland because the Central Statistical Office (GUS) does not specify the category of intellectual disability in its censuses. It should be pointed out that the surveys conducted by the GUS in which quantitative data regarding people with disabilities can be found are limited to information about the degree of disability and refer to the disabled population in the legal and biological (types of disorders) context only. Thus, it is impossible to extract information about individuals with an intellectual disability. The only source of information about the general population in Poland is the Educational Information System of the Ministry of National Education, which only collects data about individuals who are receiving compulsory education.) Moreover, for the first time in history, we are witnessing people with an intellectual disability outliving their parents and growing old together with them. It is believed that the life expectancy of people with intellectual disabilities will soon equal that of the general population [1] (pp. 91–92). This means that the percentage of dependent individuals with intellectual disabilities may also increase. (Polish society is aging rapidly. The percentage of senior citizens will continue to grow, with more than one in three Poles predicted to be 60 or more years of age by 2040, while by 2050, four out of ten Poles will be seniors. These forecasts were confirmed by the most recent report of the Central Statistical Office [2] (pp. 13, 18–19)). In recent years, the total population has been decreasing (at present, it is 38,036,1 thousand people, while at the end of 2018 it was 38,411,1 thousand). At the same time, Polish society is aging. Other challenges also exist, such as “double aging”, migration, and the weakening of family bonds. It is also worth mentioning that Poland is a country in which the family-based care model predominates. Compared to other European countries, Polish policy on the care of the dependent is very poorly developed. Due to cultural and institutional conditions, there are high social expectations regarding the involvement of the family in caring for those members that are dependent (these expectations are especially addressed to women). Social policy also mainly promotes informal care, with formal care only being supported to a very limited extent. The state delegates the responsibility, including financial responsibility, to families. In addition, while the demand for long-term care is growing, a large part of the costs is passed on to private households [3]. The marginalization of this problem is also reflected in scientific publications.

In Poland, the care of dependent people, including those with intellectual disabilities, is usually provided by informal caregivers, mainly parents. (The concept of the informal caregiver is expansive and includes family, as well as neighbors and friends, who may also take care of dependent individuals. In Poland, however, the family’s responsibility for providing care and assistance to its dependent members is a cultural norm. In the Polish literature on the subject, caregivers who are family members are called “informal caregivers” much more often than “family caregivers”. This is also justified in the law: according to the definition provided in the guidelines regarding initiatives in the area of social inclusion and combating poverty using funds from the European Social Fund and European Regional Development Fund for the years 2014–2020 [4], an informal caregiver is defined as an “actual caregiver”—that is, a non-professional caregiver who does not receive remuneration for caring for a dependent individual. Usually, this is a family member). In the literature, there is no definition of caregivers to people with intellectual disabilities; for this reason, in this article, the term caregivers to older adults is used. An informal caregiver to an older adult is considered “a person who takes care of a senior citizen for more than four hours a week and receives no remuneration for it” [5] (p. 266) (author’s translation). According to a lengthier definition, an informal caregiver is “a person who provides regular, constant, physical and/or emotional support and assistance in everyday activities to someone who is physically or intellectually disabled, mentally ill, or an older adult in a psychophysical condition that can be classified as poor” [6] (p. 19) (author’s translation). It should be noted that the concept of informal care does not actually function at the statutory level in Poland [6] (p. 19). The term was used, for instance, in the Assumptions of Long-Term Senior Policy in Poland for 2014–2020 in the context of caregivers to older adults with limited independence. In that case, an informal caregiver was defined as a person that was not a medical caregiver, taking care of a person with limited independence, such as a family member. The term has also appeared in the Guidelines of the Ministry of Development, where an informal caregiver (also called an “actual caregiver”) means an adult—who is not a professional caregiver—taking care of a dependent person (usually a family member) and receiving no remuneration for the provided care [6] (p. 19).

In Poland, informal caregivers are not registered anywhere, except for a small group of people who receive care-related benefits [7] (p. 8). This makes it difficult to estimate the number of informal caregivers. The available research shows that this function is usually performed by women, which can be easily related to cultural determinants. It can be assumed that informal care is becoming a kind of risk for caregivers: “a new category of marginalized people has emerged—informal caregivers, who are going to become beneficiaries of public social and occupational development programs in the near future. Importantly, (…) there are no studies that would show informal care as a process full of dilemmas, inner contradictions and ambivalence” [8] (pp. 23–24) (author’s translation). It is, therefore, necessary to explore this issue.

The concept of care should also be examined. This issue has aroused the interest of scholars representing various disciplines. According to many of them, the topic seems important, primarily because it concerns both the individual and communal dimensions of life. This stems, inter alia, from the increasing number of dependent individuals who need help. As a result of social changes, including demographic changes and those in the contemporary family, the problem of care affects many people. The issues, although important, are not very often addressed in research and, consequently, in the literature. This is particularly visible in research on the care of adults with intellectual disabilities. Scholars devote their attention to the care of children with intellectual disabilities; however, they seldom devote it to adults. Nevertheless, this matter is currently important insofar as it concerns two problems—the care of people with intellectual disabilities in general, and the problems of caregivers who grow older and sometimes outlive their adult children. This is a relatively new phenomenon. It sometimes happens that, as a result of aging, individuals taking care of a dependent adult become dependent themselves or find themselves having limited caregiving efficiency.

The present article focuses on the issue of assessing the situation of caregivers to adults with intellectual disabilities and, thereby, drawing attention to their problems. (Only a few publications in Polish present research on caregivers to adults with ID. This topic is often described in Poland, but in the context of children, not adults. There are also many reports on caregivers to seniors, including people with cognitive deficits).

## 2. The Place of Care in Contemporary Science

No Polish publications comprehensively and systematically present the above-mentioned care-related issues. This is particularly noticeable in the case of the literature on care activities dedicated to people with intellectual disabilities.

Naturally, the term “care” is used in gerontology and special needs education, but references to the theory are lacking. There is also no methodology, and the term “care” itself tends to be used in the colloquial sense. The review of publications revealed that there are “uncharted territories” in this area, one of them being the care of adults, including older adults with intellectual disabilities.

The term “care” is interpreted in different ways and often vaguely; it is usually understood too narrowly. It is also often used interchangeably with other terms with a similar meaning, such as protection or welfare work, or replaced with other concepts. In the narrow sense, the term refers to activity that consists in satisfying the needs of people who are, for various reasons, unable to do it themselves. Zdzisław Dąbrowski believes that “interpersonal care is based on caregiver’s (compensatory) responsibility for the care recipient and involves continuous and selfless satisfying the supra-subjective needs of the recipient in a way that involves establishing a balanced relationship of care between them and the caregiver” [9] (p. 89) (author’s translation). Limiting the definition of care to actions related solely to satisfying people’s needs and those marked by selflessness and continuity may raise doubts here. A different author, Albin Kelm, narrows down the meaning of care to actions aimed at protection from danger: “action taken with regard to people or things due to an actual or potential threat to their existence combined with limited or no possibilities for these people or things to overcome the threat unaided” [10] (p. 19).

In the author’s opinion, when defining care, one should assume that its aim is to ensure conditions necessary for health, life and development maintenance—in other words, to satisfy basic human needs and eliminate everything that poses a threat, which amounts to prevention activities.

What is indispensable for health and development (i.e., what we provide through caregiving) and what is harmful (i.e., what we try to eliminate) may vary in nature: it may be material (we provide food, warmth, and put “a roof over a person’s head”; we remove dangerous objects and eliminate dangerous situations) or psychological (we “provide” emotional support and information; we eliminate the sense of danger, loneliness, and pain). Thus, caregiving activities may be targeted both at the individual’s environment and at the individual themselves. In the second case, they may consist in developing personality traits; it is then possible to speak of care and education.

Thus, care can be understood not only as ensuring appropriate living conditions, but also, perhaps above all, as creating conditions for development.

## 3. Adults with Intellectual Disabilities

In Poland, there is an insufficient amount of precise data identifying the population of individuals with intellectual disabilities. It is assumed that intellectual disability affects approximately 1% of adults. (According to the 2021 National Population and Housing Census, the population in Poland was 38,036.1 thousand, so 1% stands for about 38,000 people). In the context of care this is a small figure, but people with intellectual disabilities have more health problems compared to the general population; therefore, they require special attention and assistance. The unique nature of intellectual disability makes the care of these people a big challenge for their families and the entire social system. The problems and needs of people with ID may be intensified by situations related to self-care deficits, somatic disorders, hindered communication, behavioral and mental disorders. It should be added that the marked demographic changes associated with the aging of Western societies are also visible in Poland, where life expectancy is increasing [11]. This also applies to people with intellectual disabilities [12,13,14]. Some studies show that this tendency will continue [14]. Life expectancy for people with intellectual disabilities increased from 20 years in 1930 to 70–74 years in 1990. This stems from the development of medical technology and modern diagnostic methods, access to specialist services, the improvement of health conditions and food quality, as well as the abandonment of institutional forms of support in favor of the promotion of housing for people with disabilities in the local community. The average life expectancy for people with mild, moderate and severe intellectual disabilities in Poland is 74.0, 67.6 and 58.6 years, respectively, compared to the average life expectancy of the general population amounting to 75.6 years for men and 81.2 years for women [15]. This phenomenon is also significant from the perspective of the functioning of families that include adults with intellectual disabilities. It quite often happens that parents act as the main caregivers to their child for several decades, until their own or the child’s death. The role of a caregiver mostly involves adjustment to a specific situation (intellectual disability) and determines almost every sphere of everyday functioning. Depending on the care recipient’s degree of disability, the family performs caregiving and protective functions, often throughout the entire time of living together. At the same time, the family acts as a legal guardian and representative.

It is also worth noting that intellectual disability has undergone considerable transformation over the years as a result of changes in scientific discourse. The shift from the medical model of perceiving disability to the social model has contributed to a paradigm change in intellectual disability research. The condition of intellectual disability is no longer treated only as a deficit, but also as a commonly accepted social fact and an element of reality.

Focusing on the defectiveness of paternalistic and segregating institutional solutions, as well as popularization of the social model of disability, has resulted in including the caregivers’/parents’ voice in the current discourse. The assumptions of inclusion, integration and normalization constitute a point of departure for opening new areas of research exploration. However, there are still too few studies on the situation of adults with intellectual disabilities and their caregivers. Therefore, it seems reasonable to continue to include the issues of people with intellectual disabilities in the scientific debate.

## 4. Aim of the Study

The main aim of the research presented in the article was to explore the situation of caregivers to adults with intellectual disabilities by identifying the themes mentioned in the responses of the interviewed caregivers.

## 5. Methods and Procedures

The study was conducted from March to September 2021 using the technique of a semi-structured interview with narrative elements. The applied instrument took the form of an interview consisting of guidelines and a set of questions. The interview scenario included guiding questions for an interviewer and the most important points regarding the issue studied. The questions were open and did not suggest any answers, which favored the collection of detailed information about the research problem. The interview conducted in the form of a conversation gave the respondents greater freedom of expression. It also allowed them to provide extra details and helped the interviewer learn about the respondents’ perspective embedded in their everyday reality. Additionally, it provided an opportunity to ask guiding and additional questions as a response to certain issues mentioned by the respondents. There were 7 main questions regarding the function of caregivers to adults with ID, their challenges and fears. The order of the questions was determined by the course of the conversation. The sampling was purposeful, and the snowball method was applied. The eligibility criteria were acting as a caregiver to a person with an intellectual disability and the care recipient’s age being above 18 years old. The sample included caregivers to individuals with a disability certificate. The disability certification system in Poland is very complicated—at present, there are two types of certifications (to receive social benefits and for other purposes), which are regulated by separate legal acts and operated by different institutions. This often results in certification documents being very general and ambiguous. Certification for the purpose of obtaining social benefits is supervised by the Social Insurance Institution (ZUS) or the Agricultural Social Insurance Fund (KRUS) for farmers and their families. Certification for other purposes is governed by the regional disability assessment boards, including those with genetic diseases such as Down syndrome, Rett syndrome and Edwards syndrome, and those with cerebral palsy. When reflecting on the situation of caregivers to people with ID, one could, of course, list the consequences of certain degrees of intellectual disability. However, this paper does not include such an analysis because this issue is beyond its scope. The author wished to show the experiences of caregivers of adults with a diagnosed ID but without analyzing the degree of disability (formally documented). Presenting the subjective perspectives of caregivers to adults with ID was the primary goal of this paper. A total of 12 caregivers to adults with intellectual disabilities were interviewed on the basis of providing their consent to take part in the study. All the interviewees lived in the Lodz Voivodeship, Poland, and the interviews were conducted in their homes. All the participants were care recipients’ parents, and most of them were women. Nine respondents were mothers. Of the three fathers in the study group, one was not a biological parent but an adoptive one (since the child was 2 years old). Most of the participants lived in a big city, and two of them lived in suburban areas. The caregivers’ age ranged from 51 to 82 years, and the care recipients were 20 to 49 years old.

The content analysis procedure consisted of five phases. The first phase involved an analysis of all the narrators’ responses to identify the raised themes. In the second phase, their codes were specified and listed. In the third phase, the codes were assigned to specific responses, and in the fourth phase, the adequacy control and correction were carried out with regard to the actions performed in phase three. The last phase consisted of grouping the codes into larger clusters—categories and subcategories. The application of a qualitative data content analysis allowed insight into and presentation of the participants’ personal perspectives on significant issues connected with providing care to an adult with an intellectual disability. This approach, falling within the ambit of the qualitative data analysis, favors the effective identification, organization and combination of diverse themes in extensive and unsorted data sets [16]. The important aim was, therefore, to gain “real-life” knowledge and an insight into the participants’ perspective. Given that informal caregivers to adults with intellectual disabilities are informal experts in assessing their situation (they have experienced several decades of engaging in care and education activities), it seemed reasonable to focus on personal experiences and perceptions. This would have been difficult to capture in quantitative research.

## 6. Results

The identification of themes concerned the caregivers’ responses to open-ended questions about their situation (How do caregivers to adults with intellectual disabilities function? What challenges does taking care of an adult with intellectual disability involve? What are caregivers to adults with intellectual disabilities afraid of?).

The table below contains thematic categories and subcategories illustrated by examples of the interviewed caregivers’ responses.

By their very nature, the fears, feelings and experiences reported in the responses are difficult to identify. They have multiple sources and can be vague. Nevertheless, based on the collected material, it was possible to distinguish thematic categories and subcategories and provide examples that illustrate them (see Table 1). It should be stressed that these categories and subcategories can hardly be regarded as fully disjunct; sometimes they overlap or complement one another.

The “everyday functioning” category included themes connected with everyday duties, their burden, intensity and routine, as well as motivation and responsibility. The interviewees constructed this category through the lens of daily ritualized practices—activities aimed at achieving specific goals. Work and duties were usually treated as tasks to be done, but also as a source to seek motivation for action. Responsibility was equated with the belief in one’s irreplaceability, i.e., perceiving oneself as the only appropriate assistance “institution.” This kind of attitude may stem from the excessive focus on the adult with an intellectual disability, overprotectiveness or “the need to be useful.” In summary, it can be said that the respondents’ everyday life seems to be a non-institutionalized aspect of family functioning.

The next category, “health”, comprised two types of responses: those concerning the health of the respondent’s adult child with an intellectual disability and those concerning the respondent’s own health. These were mainly fears associated with the concern for the care recipient’s health, which is justified due to the various concomitant diseases and complications found in people with intellectual disabilities, and with caregivers’ concern for their own psychophysical condition. Parents’ excessive involvement in their child’s disability can lead to chronic physical and mental fatigue and deteriorate the quality of their health and life. Caregivers battle against the loss of strength and stress involved in providing care to a person with an intellectual disability. They suffer from pain in their spine and joints, headaches, insomnia, dejection and experience mood changes. Some of them reported having been diagnosed with depression and anxiety disorders. It can be assumed that in some interviewees the situation led to what is known as Caregiver Stress Syndrome (CSS), a spectrum of symptoms found in people providing long-term care to dependent individuals.

The next category was called “relationships with others” and covered themes of anxiety about issues such as dependence, loss or the deterioration of relationships, as well as limited possibilities of establishing new relationships. Some of the respondents isolate themselves from interpersonal relationships. Having no time for themselves, they also do not have time for others, which can lead to a sense of loneliness and marginalization. Others, by contrast, show visible family centrism, which should be stressed is no longer a cultural value in Poland. The main characteristics of family centrism place family in the center of aid/assistance activities. Family is the reference point for most activities. The important differentiators are the holistic, systemic perception of family, moving the decision-making process from institutions to family, and considering family from the perspective of assets rather than deficits [17] (p. 133). The systemic transformation in Poland, which took place in the 1900s, influenced the sphere of family bonds—in particular, the perception of family as an important social institution. It is still treated as an environment providing support and stability, and reducing the effects of social changes on individuals, especially young ones [18]. Despite systemic, economic and cultural changes, family in Poland is still an important life value, a goal which makes life meaningful and guarantees satisfaction and happiness. Another interesting issue is the fact that the network of contacts outside the family is mostly limited to other families of people with intellectual disabilities, which may stem from the need to maintain relations only with people experiencing similar problems.

The “uncertainty” category mainly reflected general fears about the future and unstable living conditions, including finances. In the case of nearly all the caregivers, uncertainty concerned the lack of adequate provision for their adult child in case of their death. The increasing life expectancy of people with intellectual disabilities and their caregivers gives rise to numerous fears concerning the future. The respondents usually related this to the lack of an institution that could take over the care of their child. However, a visible distrust of the already functioning welfare institutions could also be reported. The sense of uncertainty also manifested itself in fears about social and material stability. The lack of such stability can lead to a sense of insecurity, frustration, loss of dignity or even exclusion from social life and stigmatization. Low living standards also decrease quality of life.

In the “time” category, two perspectives were found: one associated with a focus on the present and the other marked by a focus on the future—namely, on planning. Temporal orientation, in which the predominant focus is on the present, can have both positive and negative consequences. Philip Zimbardo and John Boyd [19] distinguished the present-hedonistic and present-fatalistic time perspectives. In the discussed research, the present-fatalistic time perspectives prevailed. They were associated with a sense of having no influence on one’s fate, which may impede the shaping of one’s life. Living with a focus on the present is associated with a lack of emotional control and stability, increased extraversion and neuroticism, internal conflicts, avoidant coping styles, impulsiveness, sensation and risk seeking, as well as alcohol and substance abuse [20,21,22]. The “planning” subcategory highlighted the caregivers’ present time orientation even more, demonstrating the impossibility of making decisions and taking actions based on the anticipated consequences and future scenarios.

The interviewees’ responses also allowed for distinguishing the “feelings” category, whose subcategories were love, satisfaction and powerlessness. The “love” subcategory was constructed by the respondents through the lens of love not only for their adult child, but also for other family members. Unconditional parental love manifested itself in the care recipient being not only loved, but also accepted. One can say that it manifested itself through functioning in an unconditional relationship in everyday life with all its challenges and burdens. Taking care of a disabled family member can contribute to strengthening bonds (marital or parent–child relations). The commonality of problems and tasks as well as mutual support builds and enhances relationships. The research also revealed that the caregivers can feel satisfaction with the fact that they provide care. This is the case when the child’s intellectual disability is treated as a blessing, a kind of “gift” and a mission to fulfill. It must also be noted that some interviewees experienced a sense of powerlessness and a lack of influence on the course of events. Powerlessness is a feeling associated with alienation, which Seeman defined as a person’s “expectancy as to whether his/her behavior can determine the outcome or reinforcement he/she seeks” [23] (p. 786). On the one hand, powerlessness among the respondents is caused by time pressure and too many duties; on the other, it is felt when they are unable to help the person they are looking after in the struggle against disease, disability, pain and suffering.

The subcategory that draws particular attention in this study is “anxiety”; it is significantly linked to other categories and subcategories. It is illustrated, on the one hand, by situations suggesting relational isolation as a “secure” condition of a person experiencing social anxiety. On the other hand, it is illustrated by situations suggesting that the lack of opportunities to use interpersonal skills leads to their atrophy. In the case of people who permanently experience social isolation through a reduction in interpersonal contacts, it can aggravate the situation. Anxiety also manifests itself in struggling with uncertainty about the future of one’s adult child with an intellectual disability when the parents are no longer able to provide them with care. The caregivers usually believe that it would be best for their child to die before they do. They are convinced that they should be the exclusive source of care and they should provide it until the end of their lives.

## 7. Discussion

The analysis of the collected qualitative material yielded an insight into the perspective of caregivers to adults with intellectual disabilities. The participants’ responses are an exemplification of the fact that universal problems find expression in individual experiences. Of course, the way of functioning results from the interaction of many individual and system factors, the support received, etc., but this issue should be addressed in further analyses.

To summarize the results of this study, it is worth noting that some interviewees perceived not only the risks and challenges, but also the positives of caring for an adult with an intellectual disability. To a certain degree, this can be treated as a sign of coping and, indirectly, as an indicator of mental health. This kind of tendency to look for the positive aspects of difficult situations is close to the concept of an optimistic style of explaining events [24]. It is also in line with the positive psychology perspective. Some researchers believe that this kind of approach makes it possible to cope with challenges more effectively, has a significant direct effect on the reduction in perceived stress, and positively impacts the sense of happiness [25,26].

However, the majority of the participants reported that providing care to an adult with an intellectual disability had a negative effect on their well-being, which was exclusively due to performing the caregiving function at the cost of their own daily life. What is visible in their responses is a kind of “addiction” to caregiving, psychophysical fatigue, and a sense of the related needs and difficulties being ignored by the broadly defined social environment (including state institutions).

Some interviewees’ statements indicated the so-called helper syndrome—namely, a caregiver’s functioning marked by a missionary nature. It consists in the caregiver deprecating other forms of conduct whose aim is other than to provide direct assistance to the care recipient. A person with this syndrome becomes a slave to the “mission” behind their conduct and perceives themselves exclusively through the lens of what they do rather than who they are. The helper syndrome is considered to be a paradoxical situation in which the helper themselves may need help.

Given the attitude towards people with intellectual disabilities, the burdens presented in this article are combined with other burdens, which can be considered in symbolic and pragmatic terms. In the symbolic dimension, caregivers feel their role being undeservedly and unjustly deprecated. Naturally, this situation does not stem from how the public evaluates their work but from contemporary people’s fear of disability, chronic disease, old age and death [27]. The pragmatic dimension refers to such burdens as a wide scope of care, communication difficulties and inadequate financial resources.

One of the practical conclusions from the above study is that the interventions should include and expand social activity aimed at identifying and supporting people taking care of adults with intellectual disabilities. They should support them in what is sometimes a very difficult psychosocial situation. It is difficult to capture and understand the mechanisms and impact of the care situation on the participants’ functioning and well-being. Nevertheless, it is necessary to intensely explore this issue and make a great effort to support caregivers’ psychophysical health, also in the context of prevention.

It should also be noted that, apart from having a diagnostic function, the results of the present study may be used in the future to develop tools for quantitative research. This kind of measure could be useful, for instance, in determining the differences in the ways caregivers’ perceive their situation. The obtained data may offer a broader and larger scale view of caregivers to adults with intellectual disabilities.

## 8. Conclusions

The high tendency of many Polish families to look after their loved ones is usually treated as a positive sign of bond, dedication and, sometimes, intergenerational solidarity. What is less readily noticed is the fact that individual decisions are sometimes forced by various circumstances. The risks and hidden or delayed individual, social and economic costs are usually overlooked. Few studies, especially studies conducted in Poland, focus on similar issues. Of the available publications, psychological research on the stress and parental attitudes of mothers and fathers of adults with ID should be noted. Dorota Suwalska-Barancewicz and Alicja Malina [28] showed that stress is a factor which is constantly present in the daily functioning of families with a disabled family member, and that it affects the process of adapting family members to this challenging situation. They also revealed a correlation between parenting attitudes such as setting expectations, a lack of consistency, protection and the aspects of stress: intrapsychic, external and emotional tension. The results of the research presented herein confirm the thesis about the stressful nature of caring for a person with ID and its emotional consequences. Laurence Taggart, Maria Truesdale-Kennedy, Wniosko Ryana and Roy McConkey [29], who studied caregivers in the UK, also concluded that aging caregivers need emotional, informational and practical support. They also state that such support should determine the direction of social policy development in other countries. Similar conclusions were formulated by McKenzie and McConkey [30], who also pointed to the necessity of providing different types of support to caregivers in South Africa. It is also worth adding that the analysis of the literature on the care of people with disabilities and the elderly shows that many researchers have reached similar conclusions. This is evidence that caring activities are a great burden [3,31]). In summary, based on the present study, it can be concluded that the costs on the caregiver’s part include, above all:

- The risk of neglecting one’s own health as a result of focusing on the care recipient only (an adult child with an intellectual disability);
- The risk of physical fatigue, including the risk of injuries associated with caregiving (e.g., injury as a result of lifting the person one is taking care of);
- The risk of chronic stress, mental “burnout,” anxiety disorders, depression and other mental disorders;
- The risk of limited social contacts and, as a consequence, social isolation;
- The risk of material exclusion (poverty) and, as a result, social exclusion of the family members who provide care to an adult with an intellectual disability.

Apart from affecting the individual’s well-being, the existence of these individual risks carries socioeconomic costs. Counteracting these risks and costs would require a reorientation of the existing support system. Currently, it is mainly based on cash benefits (low and selective), with a shortage of non-financial assistance. Non-financial support includes different services and assistance, so as to help combine care with occupational work. In the Polish social assistance system, these non-financial forms of support are the least developed. On the systemic level, there are no solutions like those mentioned in the literature and functioning in some developed countries. They include short-term relief (respite care), information, counseling and training for caregivers, commonly available daily support instruments and instruments supporting the effective organization of professional work and long-term care. Such support is insufficient—there is no possibility of using respite care (short term-relief) or psychological and therapeutic caregiver support. Legal regulations are also missing—there are no labor law regulations that would adjust working conditions to caregivers’ needs and possibilities.

The new approach to the care of adults with intellectual disabilities should seek a greater balance of responsibility for organizing, financing and providing care between the individual, their family and the community (including the state). Families providing care for their members should not meet with passivity on the part of the state, but rather with its support. Institutional care should be accompanied by actions promoting the dependent person’s integration with the social (and family) environment and by creating conditions at the institutions that would be similar to those at home and in the community. The network of support should be complemented with day centers, formal services provided in domestic settings and services targeted at informal caregivers.

The care of a dependent person with an intellectual disability should be viewed on a broad human spectrum. It should be considered as a unique situation of those who remain under permanent care provided by family members, those who live alone and those whose loved ones try to combine caregiving with their own private lives.

## Figures and Tables

**Table 1:** Thematic categories and subcategories.

Category	Subcategory	Examples of Responses
Everyday functioning	Routine	Washing, dressing, eating, drinking, day after day…(W62,D31). (The narrators were coded as follows: caregiver’s sex (woman or man) and age and care recipient’s sex (daughter or son) and age). All my days are very much alike (W59, D32). There are a lot of routine everyday activities to do (W73,S42). The same activities, day after day (W51,D29). From the morning until the night, there is work to do all the time…(M63,D33). Basic care all the time, the everyday sort of work, such as washing, and so on (W76,D41).
	Multitude of duties (the burden and intensity of care)	I am at her beck and call, and I react to everything almost at once (W50,S20). Hard work, that’s what I would call it—comparable to working in a mine (W69,S42). There are so many things to do every day that it is difficult to embrace all of them with your mind with your body too (W51,D29).
	Motivation	Sometimes it was difficult, because cooperation with him was far from easy, but I always believed that things would get better, and this gave me the strength (W76,S41). The worst thing to happen would be to lose hope, because it has always kind of motivated me, and it still does…(W66,D32) For me, God is the motivation… Then I know that I want to do this, not that I have to…(M71,D36).
	Responsibility	I look after him because he is my child and I am responsible for him, very responsible… If I didn’t, who would? (W51,29) Sometimes it is difficult with her, but then I tell her: remember that I am the only person you have and that I am responsible for you (W62,D31). I have to be alert all the time… After all, I am responsible for her… 100% responsible (W72,D47).
Health	Health of a person with ID	It is also a constant struggle for her health (M71, C36). He is sickly; there are so many diseases in that frail body of his (W50, S20). She was born sick and is ill all the time… I’m worried (W66,D38).
	Respondent’s health	I’ve been in treatment for depression for many years, and I think this is because I’m overburdened with caregiving duties… I don’t sleep at nights, I walk and think…(W72,D47). To be frank, I sometimes had bad thoughts and had to be treated…(W51,D29). My health is another story. Things are not good, but this is hardly surprising after so many years of neglect, I have only myself to blame… And now my spine, my joints, everything is falling apart… I can’t sleep, either (W62, D42). I have developed a neurosis, a severe neurosis (W59,D32). The worst thing is the headaches, let me tell you. I’ve had them for years (M71,D36). When I look at my life I can see a lot of sorrow and little joy. This has affected my health, so now I feel 80 instead of 50 (W51,D29).
Uncertainty	Fears about the future	It is the future that is a problem; I don’t know what will happen and how it will be (M82,S49). I look at myself, my husband, and my son and I can see fear, a fear for our and his future (W76,S41). We have been looking after her for many years, and I know she needs this, but we are already old, and this worries me… It worries me very much (M71,D36).
	Unstable living conditions	But there is not enough money—transport, food, medicines, rehabilitation… Everyone knows it all costs money (W69,S42). There is never enough money when only one person works (W51,D29). My husband is going to retire soon, which is both good and bad news… He will spend more time at home, but are we going to manage financially? It will be rather hard (W59,D32). My son is so dependent that he is forced to rely on me completely, and I’ve been working on and off, so it is hard for us… Such an uncertain existence…(W50,S20)
Relationships with others	Dependence	Sometimes I have an impression that we are glued to each other (W76,S41). At times I feel as if I were my daughter’s slave (M63,D33). I guess he is actually entirely dependent on me (W69,S42). I am stuck, I have no freedom or liberty… (W66,D38)
	Loss or deterioration of relationships	My husband, my son, and I make up the circle of my friends, acquaintances, everything… (W76,S41). Even when we go on holiday together there are only the three of us, who else could possibly go… So it is hardly a holiday for me; only the place changes but the duties remain the same (W62,D31). I have no time for friends… In fact, I have no friends because I have no time or place to meet with them…(W50,S20). I’m a little sorry that I was unable to take care of my relationships with other people. At first I guess I was ashamed when he was little, and then we went out of touch… And so it has remained… (W73,S42). I must say that my interpersonal skills have disappeared, that’s how I see it… And anyway, where could I possibly meet anyone? (W66,D38). I don’t get out much, you know. Sometimes I have a word with my neighbor or with the postman who brings my pension, but that’s all (W76,S41). Our friends are mainly people from our community…(W69,S42). Sometimes we meet with the mothers… During rehabilitation periods, for example (W59,D32).
Macro level	Political and economic situation	Total disappointment with the system, politics, politicians, and everything…(M63,D33). I’ve been to the Municipal Social Assistance Center many times, and to what effect? What kind of assistance is that? I no longer even feel like laughing…(W62,D31). I do need support, but I really don’t know where to look for it. I think this system is not working at all…(W76,S41). Our leaders are not doing anything about it, and people are suffering…(W59,D32). People like us are ignored in this country… and our life is getting more and more miserable (M63,D33). The disability assessment system is a real disaster…(W62,D31). Everything costs a lot of money… and where am I to get the money from? And I’m only talking about the absolutely necessary things, not whims or fancies…(W59,D32).
Time	The present Plans	Day after day… here and now… one does not look back…(W62,D31). I live a day-to-day existence and I don’t think about what tomorrow brings (W59,D32). Today things are as they are… and tomorrow we will see (W50,S20). Sometimes it is even difficult to simply dream about something, because you are unable to predict what will happen tomorrow (W66,D38). You know, we never make any plans, we’ve got used to this and we have learned that in our case planning makes no sense (M63,D33). With this kind of lifestyle and this kind of care, it is not even possible to make plans for the next week, let alone for years ahead (K62,D31). I don’t have time for myself; actually, I don’t have time for anything, and I definitely don’t have it for pleasures, so I don’t even plan them (W51,D29).
Feelings	Love	My son constantly shows me what is important in life… He makes me realize the meaning of true humanity, love... (W76,S41). She has taught me unconditional love, the kind of love a child deserves. Not for living up to our expectations but simply for being there… being as she is (W72,D47). I love him as he is, even though sometimes I’m angry or mad at him… but, after all, he is my only child (W50,S20). My husband is not only the love of my life but also a great support to me (W69,S42). Without my beloved daughter my life would have been lost long ago… She helps me and gives me so much, my dear one…(M71,D36).
	Satisfaction	To go through it all myself has been a great experience and happiness… although not everyone is capable of looking at it that way. God wanted it that way (M82,S49). We can all derive joy and strength from him… He is a good child, a kind of gift, one might even say (W76,S41). We have put lots of work into him.—Apparently, God knew what he was doing by giving Paul to us. He doesn’t send such children accidentally. He sends them only to those who will bear it, and we’ve been coping (W73,S42).
	Powerlessness	There are situations in which I am helpless and unable to help her (W73,S42). A terrible feeling of powerlessness comes over me sometimes… a kind of terrible powerlessness… (W51,D29) Sometimes I give up, but what can I do? Nothing at all…(W59,D32)
	Anxiety	Sometimes I dream about living peacefully and not having to fear for tomorrow or the day after tomorrow (W66,D38). I’m afraid of large groups of people… I think I’ve become unaccustomed to people because we rarely go out (W62,D31). You know, the worst thing is the constant fear for virtually everything… for what it will be like if I don’t manage (W72,D47). I no longer like to go or travel to places, I avoid such situations and I’m afraid… It feels good at home (W62,D31).

Source: the author’s research.

## Data Availability

To protect the privacy of the study participants, confidentiality standards were applied both in the case of the data collection and at all stages of analysis. No personal data were collected in the study, apart from age and gender. The Investigator will preserve the confidentiality of the participants taking part in the study and fulfil transparency requirements under the General Data Protection Regulation. Only the Investigator has access to the personal data of the participants and to the final dataset.
